# “The reality is that on Universal Credit I cannot provide the recommended amount of fresh fruit and vegetables per day for my children”: Moving from a behavioural to a systemic understanding of food practices [version 1; peer review: 2 approved]

**DOI:** 10.35241/emeraldopenres.14062.1

**Published:** 2021-02-18

**Authors:** Maddy Power, Katie J. Pybus, Kate E. Pickett, Bob Doherty

**Affiliations:** 1 Health Sciences, University of York, York, UK; 2 The York Management School, University of York, York, UK

**Keywords:** poverty, food insecurity, food banks, diet, public health, food poverty, childhood food poverty

## Abstract

**Background:**

Evidence suggests that people living in poverty often experience inadequate nutrition with short and long-term health consequences. Whilst the diets of low-income households have been subject to scrutiny, there is limited evidence in the UK on the diet quality and food practices of households reporting food insecurity and food bank use. We explore lived experiences of food insecurity and underlying drivers of diet quality among low-income families, drawing upon two years of participatory research with families of primary school age children.

**Methods:**

We report on a mixed-methods study of the relationship between low income, food bank use, food practices and consumption from a survey of 612 participants, including 136 free text responses and four focus groups with 22 participants. The research followed a parallel mixed-methods design: qualitative and quantitative data were collected separately, although both were informed by participatory work. Quantitative data were analysed using binary and multinomial logistic regression modelling; qualitative data were analysed thematically.

**Results:**

Lower income households and those living with food insecurity struggle to afford a level of fruit and vegetable consumption that approaches public health guidance for maintaining a healthy diet, despite high awareness of the constituents of a healthy diet. Participants used multiple strategies to ensure as much fruit, vegetable and protein consumption as possible within financial constraints. The quantitative data suggested a relationship between higher processed food consumption and having used a food bank, independent of income and food security status.

**Conclusions:**

The findings suggest that individualised, behavioural accounts of food practices on a low-income misrepresent the reality for people living with poverty. Behavioural or educational interventions are therefore likely to be less effective in tackling food insecurity and poor nutrition among people on a low income; policies focusing on structural drivers, including poverty and geographical access to food, are needed.

## Introduction

Food insecurity, the “limited or uncertain availability of nutritionally adequate and safe foods or limited or uncertain ability to acquire acceptable foods in socially acceptable ways”^1^, has increased across Europe and in the UK since the 2008 economic crisis^2,3^. Food insecurity was present among a large minority of the UK population before COVID-19^4^, however the pandemic, and the associated economic fallout, has precipitated a sharp increase in food insecurity in the UK^5,6^. In July 2020, roughly 16% of adults – equivalent to 7.8 million people – reduced meal sizes or skipped meals due to insufficient income for food; this figure, which remained stable between April and July 2020, is roughly double the rate of food insecurity before COVID-19^7^.

Food insecurity in North America has been found to be associated with poor diet and food insecure adults report lower intake of fruit, vegetables and dairy compared to food secure adults^8,9^. Emerging research on food insecurity in the UK suggests a similar relationship with diet^10,11^ – analysis of the International Food Policy Study by Yau *et al.*^11^ found that food insecure adults have a lower probability of consuming fruits and vegetables than food secure adults, and a higher probability of reporting unhealthy diets.

Inadequate nutrition is well established to be a particular concern for public health^12,13^. An unhealthy diet, defined as one which is high in fat, sugar and salt, and low in fruit and vegetables, can have long-term negative health consequences, especially for children, and makes a major contribution to health inequalities^8,14–18^. People living on low incomes are more likely to become obese, suffer from heart disease or type 2 diabetes, or experience complications/secondary health problems relating to obesity, heart disease and diabetes^19–23^. Reflecting this, in 2005, food related ill health was found to be responsible for around 10% of morbidity and mortality in the UK, costing the NHS roughly £6 billion annually^24^. Inadequate nutrition among people living in poverty has been the subject of much contention, with an emphasis on individualised narratives of ‘poor choices’ and a lack of knowledge or education, whilst lived experiences and the structural drivers of food practices have arguably been relatively neglected^25,26^.

In the UK, this debate has been most recently reignited by campaigns to extend free school meal provision and Healthy Start vouchers during the pandemic^27^, which have met with some resistance. However, the relationship between food insecurity and diet in the UK remains under-researched; there is limited evidence on both the food practices of households reporting food insecurity and the lived experience, including the socio-psychological impact, of maintaining a healthy diet in the context of food insecurity.

The rise in food insecurity over the past decade has been accompanied by a sharp increase in the number of food banks providing emergency food support^28^; the Trussell Trust, the largest network of food banks in the UK, distributed 25,899 food parcels in 2008–2009 compared to 1,900,122 in April 2019 to March 2020 – an extraordinary rise even before the onset of COVID-19^29^. The drivers of food bank use and the demography of those using food aid services has been discussed extensively^30–32^, but similar to food insecurity, there remains limited understanding of the diet quality of food bank users or how people using a food bank view the nutritional content of the food they receive. The absence of research in this area is of particular urgency in light of the sharp rise in the use of food banks since March 2020 by people experiencing poverty and income shocks^33^), and nutritional concerns about the content of food bank parcels^34^ which pre-existed COVID-19.

### Research approach and aims

This article draws on two years of mixed methods participatory research with people living with poverty and at risk of food insecurity, as well as service providers responding to poverty and food insecurity. This study placed experts-by-experience, as both service providers and service users, at the centre of the research design and delivery. In so doing, it sought to open up a space for the emergence of alternative narratives of food, poverty and food consumption, whilst simultaneously prioritising community concerns around food insecurity and food bank use, and building community capacity in confronting food insecurity and poverty.

The aims of the research were motivated by service providers and services users, who considered the diet quality and consumption patterns of people living with food insecurity and poverty to be a key concern in the area. As such, the aims were as follows:

1. To assess how food insecurity and food bank use impact food consumption and diet quality among households with young children.
2. To conduct research on household food insecurity and food practices that reflects community priorities to, in turn, inform local responses to food insecurity and nutritional inequalities.

## Methods

### Study design and setting

The study consisted of a survey and four focus groups (outlined below). It was initiated and co-produced by members of the York Food Justice Alliance (YFJA), a multi-sector organisation encompassing people with lived experience of food insecurity, community food aid providers, local authority representatives, local charities, academics and other relevant stakeholders focused on tackling food insecurity in York.

Accordingly, the study took place in York and prioritised questions of greatest importance to YFJA stakeholders – notably food choices and diet quality among low-income households – and the sample (households with young children) reflects an identified area of local need. Although York, with a population of 210,000 people and situated in the north of England, is an affluent city compared to the wider Yorkshire and Humber region, there are considerable inequalities and hidden poverty. The York Fairness Commission has observed that there is an ‘Advantaged York’ and a ‘Disadvantaged York’^35^ and, in 2017–18, over 4,000 people in the city used a Trussell Trust food bank, including over 2,600 children^36^.

#### Ethical approval

Ethical approval for the study was provided by the University of York Health Sciences Research Governance Committee on 06.07.2018. All participants provided consent for publication of their data. All data is anonymised.

#### Survey

The survey was designed collaboratively with members of YFJA and aimed to collect the appropriate evidence to inform local responses to food insecurity and poverty (see *Extended data* for a copy of the survey^37^). Experiences of food insecurity were identified using a validated two-item measure^38^, derivative of the 18-item US Household Food Security Survey and widely used in clinical settings. Given the need to cover a range of topics important to YFJA stakeholders, the two-item measure allowed for robust assessment of household food insecurity^38^ whilst limiting the number of survey questions overall. Demographic characteristics such as household type and income were measured using existing Office for National Statistics categories. Fruit and vegetable consumption was self-reported and assessed via the question, ‘How often do you and your household eat fruit and/or vegetables?’ with possible responses including ‘Less than once a week/One to three times a week/Once a day/At least twice a day’. Consumption of processed food was also self-reported, assessed via the question, ‘How often do you and your household eat processed food and/or ready meals?’. These two questions were developed through a consensual process with YFJA members to reflect community interests and priorities. In addition, the survey included a single question to assess self-reported food bank use, ‘Have you or another member of your household ever used a food bank?’. A free text response box was provided at the end of the survey with the question, ‘Do you have any further comments on food in York?’ to explore wider food experiences, including issues of food access.

Adult members of households with primary school aged children (4–11 years) in York were surveyed about their experiences of food quality, food insecurity and food bank use. All 63 primary schools in York were invited through the YFJA network to take part in the study and 25 agreed to participate and to distribute the survey to parents.

Schools were approached by YFJA members in the first instance with verbal and written explanations of the study. Once participation was confirmed, paper copies and an electronic link to the survey were provided and disseminated to the caregivers of pupils in each school by letter and/or email. The survey was also shared via social media channels, such as Facebook. The text of the survey was accompanied by an information sheet documenting the purpose of the study, data storage and use, and the process of consent. Written informed consent was obtained from all survey participants. The survey was open for participation from November 2018 to February 2019.

#### Focus groups

Negotiation of food quality and food quantity in contexts of low income and food insecurity was further explored in four semi-structured focus groups held in January 2019. The author worked with community groups in York and members of the YFJA to identify and recruit parents and carers living on a low income; participants were either approached directly by a member of partner community groups or informed about the focus group via leaflets distributed in community venues, including the community venues in which the focus groups were held. Participants self-identified as a parent or carer living on a low income and choose to participate in the focus groups. The focus groups were held in a familiar location, such as a community centre or a low-cost, community café, and lasted between one and two hours. To ensure confidentiality, the focus groups were conducted in a private room or setting in the community venue. The focus groups were moderated by the first author and a research assistant, with experience of moderating group interviews. In line with the preferences of participants no recording equipment was used; instead, written notes were taken. The topic guide (*Extended* data^37^) was produced collaboratively with members of YFJA, constructed to explore the lived experience of food and diet in contexts of poverty and low income. Confidentiality and informed (oral) consent were maintained throughout and all data was anonymised during transcription and analysis.

### Strategy for analysis: survey and focus group data

The research followed a parallel mixed methods design, in which the qualitative and quantitative data were collected separately^39^, although both were informed by discussions using a participatory approach. Findings were triangulated at the analysis stage using a convergence approach^40^, with qualitative findings used to explain and expand on the quantitative data.

Following collection of the surveys, a dataset of quantitative responses was created and uploaded into Stata 16.1 for analysis. Responses to the food insecurity questions were merged to create a single, binary food insecurity variable, according to established methods^41,42^. To enable adequate analysis from the response data obtained, we recoded processed food consumption into a binary variable: eat less than once per week/eat more than once per week. Similarly, fresh fruit and vegetable consumption was recoded into three categories: three times a week or less, once daily, twice daily or more. Quantitative data were analysed using binary and multinomial logistic regression modelling. Free-text responses were collated and analysed using a thematic analysis framework^43^. MP and KJP separately reviewed the data and proposed categories were formulated. Thesecategorisations were discussed until a consensus was reached.

Focus group transcripts were coded and analysed thematically by MP and a research assistant to elicit common themes related to the research aims. Data categorisations were discussed until a consensus was reached.

## Results

### Quantitative survey data

The survey was disseminated by schools and shared through social media using both an electronic link and hard copies. As a consequence of the multiple methods used to distribute the survey, it is not possible to provide an accurate overall response rate. Nevertheless, the response rate from paper copies of the survey distributed via primary schools was 11%, showing the value of pursuing dual (online and offline) methods of dissemination. Overall, the survey achieved 612 individual responses, with 136 free-text responses.

Demographic characteristics of the sample, reported in Table 1, demonstrate that the majority of households contained two adults (n=463, 75.65%) and two children (n=329, 54.83%). There was an overrepresentation of higher income households: 43.57% (n=261), having an annual total household income of over £38,399. Of our respondents, 23.37% (n=140) reported experiencing food insecurity, whilst 7.54% (n=46) stated that they or a member of their household had used a food bank.

#### Household composition and diet

The results (Table 2) demonstrate that households with an income above £28,000 per annum have a greater likelihood of eating fresh fruit and vegetables daily compared to three times a week or less, and households in the highest income group were 6.35 times (95% CI: 3.21, 12.57) more likely to eat fresh fruit and vegetables twice a day or more, than households with the lowest incomes. There was no difference in fruit and vegetable consumption in the lowest two income groups, 38.03% of households earning less than £16,100 per annum and 38.89% of those earning between £16,100 - £21,249 ate fresh fruit and vegetables three times a week or less. We did not find an association between the number of adults or the number of children in a household and the frequency of fresh fruit and vegetable consumption. Whilst adding the number of children and adults in the household to the models did slightly modify the relationship between income and fresh fruit and vegetable consumption (models 1.b and 1.c), these factors had little impact on either the strength or the direction of the association. We did not find an association between income, adults or children in the household and frequency of processed food consumption (Table 3).

#### Food insecurity, food bank use and diet

Respondents who were food insecure in our sample were half as likely as those who were food secure to report eating fresh fruit and vegetables three times a week or less, compared to once daily or more (OR: 0.46: CI: 0.28, 0.76). This association was partly accounted for by income, which nullified the relationship between food insecurity and eating fresh fruit and vegetables once per day, but only modified the association between food insecurity and eating fresh fruit and vegetables at least twice daily (OR: 0.42: CI: 0.24, 0.75), see Table 4. There was a strong negative association between having used a food bank and frequency of fruit and vegetable consumption, but this relationship appeared to be accounted for by the addition of income and food security status to the model.

We found a weak, positive association between being food insecure and a greater likelihood of processed food consumption, but the relationship between having used a food bank and processed food consumption was much stronger. These respondents were over two and a half times more likely to describe eating processed food more than once per week (2.67: 1.41, 5.05), compared to less than weekly (Table 5). Neither food insecurity, income, nor a combination of the two, were able to account for this association (model 4.d).

### Qualitative survey data and focus group data

The focus groups included 22 participants, across four focus groups (7, 7, 5, 3), the majority of whom were female (n=19). All participants had children and all self-identified as living on a low income. The qualitative data across the survey and focus groups was rich with themes relating to experiences of food on a low income. In view of the focus of this paper, we concentrate our analysis of the qualitative data on experiences, challenges and – largely systemic – barriers to healthy eating on a low income. The other findings from the qualitative data are reported elsewhere^44^. We compare key themes across the survey and focus group data, highlighting points of divergence where they arise.

#### Theme 1: Barriers to healthy eating on a low income

Participants in the focus groups and survey discussed at length and with great frequency the multiple barriers to maintaining a healthy and varied diet on a low income. It is well established that a low income is a key barrier to accessing sufficient fresh fruit and vegetables^19^, and this was highly evident in our qualitative data.

The reality is that on Universal Credit I cannot provide the recommended amount of fresh fruit and vegetables per day for my children and I go without more times than not so they can have my share. (survey)

Equally prominent was concern about the high cost of fresh meat and fish, and perceptions of being priced out of these foods and/or replacing these forms of protein with cheaper options:

I am a one-parent family and work part time, however I’m fortunate enough to not have to worry about food and fuel. I shop carefully and sacrifice other things to be able to buy fresh and non-processed as far as possible. However, I cannot afford to buy fresh fish or meat as often as I would because of the high cost, and therefore use lentils, pulses, beans and nuts as a regular source of protein. (survey)

Meat is just too expensive to have every day and we eat lots of pulses. (survey)

Participants acknowledged processed food was often more accessible than ‘healthy’ options because of its lower cost:

Healthy food is more expensive. The supermarkets always have deals on processed/convenience food. (survey)

Healthy food is expensive and unhealthy food is cheap. (focus group)

Awareness of being priced out of nutritious and fresh food because of low income reinforced the stigma of living with poverty and was a very visible and everyday example of socio-economic inequality, particularly for parents and carers who were keen to ensure their children had access to a healthy diet:

It’s not nice to feel you can’t buy food that is healthy/ better because it’s more expensive. (survey)

Access to healthy and fresh food was further constrained by geographic access and availability. The availability of fresh and healthy food in local shops was perceived to be poor, but the cost of travelling to large supermarkets, where the quality and diversity of food may be better, was considered prohibitively expensive:

Local supermarkets are mainly convenience and processed food. There is not enough fresh produce to choose from, not enough fresh fish and too much farmed fish. (focus group)

It would help to feed my kids healthily if smaller shops that I walk past would sell good quality, reasonably priced fresh fruit and veg’. (survey)

Access to fresh produce is limited here but the cost of buses is prohibitive. (survey)

A combination of poor geographic access, high transport costs, low income and the high cost of nutritious food thereby severely constrained the accessibility of a healthy diet, despite very high awareness of its components.

#### Theme 2: Management strategies to attempt to achieve food security, including food quality, on a low income

Participants in the survey and focus groups described attentive and time-consuming shopping strategies employed to maintain a healthy diet for themselves and their children. This included attending multiple varied outlets (“shopping around” – focus group) to search for low prices and “offers” (focus group); visiting budget supermarkets; and buying items at the back of the shelf with the longest date mark. Among a significant minority of participants, buying secondary produce – “wonky” fruit and vegetables; out-of-date, reduced-cost items; and end-of-the day unsold fruit and vegetables in markets – and eating vegetarian food rather than (expensive) meat and fish were important strategies in purchasing adequate food on a low income. Less common, but still discussed strategies involved shopping seasonally, replacing expensive ingredients with cheaper alternatives, and parents reducing their own consumption to ensure adequate good quality food for their children.

There were inherent disadvantages to such management strategies. Visiting multiple shops to find the lowest prices could be time-consuming, highly stressful and challenging with young children:

I spend a lot of time checking prices and buy items from different supermarkets taking advantage of offers. It's quite exhausting! (survey)

The food is getting more expensive and I am always anxious to go to the shops and see how much I spend as it looks more all the time. Going to shops creates a lot of stress. I use my credit card to pay for food and hope I will have enough money to cover it every month. (focus group)

The need to navigate high and rising food costs, whilst caring for young children and managing already extremely tight household budgets, added significantly to the pre-existing stress, anxiety and stigma of life on a low income^45,46^.

#### Theme 3: Food aid and healthy eating

The quantitative survey data showed a clear association between use of food banks and higher consumption of processed food, independent of income and food security status. The qualitative survey and focus group data, however, pointed to a more nuanced picture. The quantitative data indicated a relatively low (20%) use of food banks among people experiencing food insecurity (see also^44^); this was re-emphasised by the qualitative data in which (across the 158 qualitative respondents) only three participants discussed currently or previously using a formal food bank. Nevertheless, there was some evidence of the use of *informal* food aid, including community cafes and informal food banks (places in which food is freely available in a specific area of the building for anyone to take). Focus group and survey participants described positive experiences – contrasting with negative descriptions of formal food banks, described by ourselves elsewhere^44^ – and particularly valued the fresh fruit and vegetables often available through informal food aid, stressing its importance in improving the diet quality of themselves and their family:

We only eat fresh veg and fruit because of the use of free food at the community cafe. (survey)

We need more community cafes, ones that are large and welcoming enough for families. (focus group)

The impact of food aid on improving access to nutritious food was, in this study, highly variegated and appeared to be contingent upon the type of food aid in question.

## Discussion

### Main findings

Our quantitative findings suggested that those on lower incomes and who are food insecure may struggle to access a level of fruit and vegetable consumption that approaches public health guidance for maintaining a healthy diet. This finding was corroborated by the qualitative data in which parents and caregivers clearly articulated their desire for a healthy diet, however the high cost of fresh fruit and vegetables, coupled with low incomes, was described as a key barrier to eating healthily. The location of supermarkets outside of the city centre, accompanied by high food costs in local convenience stores – consistent with the premise of a food desert^47,48^ – and high transport costs further constrained food options. Although not addressed by the quantitative survey, there was also considerable evidence in the qualitative data of difficulties affording fresh meat and fish due to high costs.

Within the limitations of the questions included in the survey, our data did not suggest that people living with food insecurity and poverty are more likely to eat processed food. Indeed, there was widespread acknowledgement that processed food was often more accessible than “healthy” options because of its lower cost, but also detrimental to health and consequently avoided. In contrast, the qualitative data evidenced attentive, time-consuming and often stressful shopping strategies employed by participants to maintain a healthy diet for themselves and their children.

Whilst base sizes may be too small to allow for robust conclusions, the quantitative data did indicate a relationship between higher processed food consumption and having used a food bank, independent of income and food security status. The qualitative data, however, suggested that the relationship between diet quality and the use of food aid may be more nuanced. In particular, there was evidence that informal food aid, often providing free fresh fruit and vegetables, enabled some low-income families to maintain a healthy diet in the absence of an adequate income that would allow them to purchase such a diet.

### Discussion in relation to the literature

Echoing previous literature^49–52^, parents in this study possessed the knowledge and ability to make healthy decisions about the diets of themselves and their children, but a range of structural factors, most prominently their income and their food environment, severely constrained these decisions. As identified by Attree^51^ in a systematic review of qualitative studies on the lived experience of poverty, food and nutrition were important facets of managing poverty for low income families. Parents, and especially mothers, strategically adjusted to living on a low income by adopting a number of approaches. Whilst the strategies employed to get by on a low income, such as cutting back and making do, appeared to become second nature, the stigma of poverty and sense of exclusion from ‘ordinary living patterns’^53^ was keenly felt. Choice around food purchases was experienced within externally imposed limitations; real and meaningful choice did not exist for parents living on a low income, for whom food was a vehicle for social exclusion rather than inclusion.

Food insecurity is predominantly a consequence of poverty, as identified by multiple studies^3,54^, including our own^44,55^. As such, it impacted diet quality in similar ways to low income. Whilst this study did not focus in detail on food bank use, the findings reflect those of Puddephatt *et al*.^56^ who, in a qualitative study of food bank users in Liverpool, found income to be the most salient factor influencing participants’ food choices. In this latter study, all participants reported a constant struggle to afford food; food decisions were primarily based on cost and most participants valued eating healthily, but could not afford to do so^56^.

### Strengths and limitations

This study is one of the first to adopt a participatory process to explore food insecurity, food bank use and food practices among a UK population. By so doing, it reflected the concerns of local stakeholders in its research focus – food practices and diet quality among low-income families with young children – strengthening community cohesion and instigating community action to improve the quality of food in local community food aid. The co-produced research underpinned meaningful policy impact, precipitating the establishment of a Food Poverty Scrutiny Group within the local authority, a key demand of the YFJA.

The mixed methods approach created a broad and deep understanding of food insecurity, food bank use and food consumption, highlighting the wider structural context of food insecurity and food practices; the absence of agency low-income families may have in decision-making around food; and the relational dimensions of food experiences.

Nevertheless, the study has some weaknesses. The questions asked in the survey were based upon a collaborative process reflecting community priorities around food insecurity. Although wherever possible questions were based upon validated and established measures, the question relating to processed food consumption, for example, was not and therefore comparisons with other studies should be treated with some caution. A more robust method of assessing diet quality in the sample would have involved using a food frequency questionnaire, but this was precluded by the need to develop a relatively short survey and by the participatory nature of the research, which meant that questions were only included where these were deemed to be a priority by consensus across different stakeholders in the group. Future research may wish to focus on the issues identified here in more depth.

The research was conducted in a single city, and one with a particular demography; comparability may therefore be limited to similar towns and cities rather than to the UK as a whole. The sample includes families with young children only and both the quantitative and qualitative sub-studies were opt-in, potentially leading to an under-representation of low income and marginalised groups, and an over-representation of more affluent groups – indeed, there was some indication of this in the survey sample demographics. Whilst the qualitative sample is relatively large, including 22 focus group participants and 136 free-text qualitative responses, the sample for the quantitative analysis is small; in particular there are low numbers of people using a food bank as part of the overall sample (N=45) and people reporting food insecurity (N=140), limiting analysis of these groups.

## Conclusions

This study shows that whilst many families exist on inadequate diets, the detrimental consequences in terms of social, emotional and nutritional wellbeing are concealed and ‘individually embodied’ rather than considered and addressed as part of broader systemic inequalities^57^. Broadly, our study demonstrates that the diet of low-income families is dictated primarily by a lack of affordability of certain food groups, rather than by choice – a form of enforced thrift. Participants in our study were acutely aware of both the constituents of a healthy diet and their social exclusion from not being able to access this.

These findings suggest that individualised, behavioural interventions are likely to be ineffective in improving food security among parents with young children and that policies focusing on addressing the structural drivers constraining a socially acceptable standard of living and eating are needed. Examples may include ensuring all those in work have access to the living wage and secure employment, as well as changes to the social security system to ensure that families can expect their income to rise in line with living costs. Given that 13% of the UK population experienced food insecurity before COVID-19^4^ – a figure that has increased sharply as a result of the pandemic^5,6^ – an income-based approach will ensure that a larger number of families are able to afford adequate nutrition. In turn, some of the long-term individual and public health effects of poor diet may be averted for a substantial section of the population.

## Figures and Tables

**Table 1:** Sample demographic characteristics.

Demographic characteristics	N (%)
**Annual household income**	
Less than £16,100	71 (11.85)
£16,100 - £21,249	72 (12.02)
£21,250 - £27,999	78 (13.02)
£28,000 - £38,399	117 (19.53)
More than £38,399	261 (43.57)
*Total*	599 (100)
**Adults in household**	
Single adult	117 (19.12)
Two adults	463 (75.65)
Three adults or more	32 (5.23)
*Total*	612 (100)
**Children in household**	
One child	161 (26.83)
Two children	329 (54.83)
Three children or more	110 (18.33)
*Total*	600 (100)

**Table 2:** Demographic characteristics and fresh fruit and vegetable consumption.

Fresh fruit and vegetables base: three times a week or less		Model 1.a	Model 1.b	Model 1.c
**Annual household income**				
Once per day	Less than £16,100	-	-	-
	£16,100 - £21,249	1.05 (0.47,2.33)	1.11 (0.48, 2.52)	1.08 (0.48, 2.41)
	£21,250 - £27,999	1.11(0.48, 2.55)	1.08 (0.45, 2.55)	1.19 (0.49, 2.90)
	£28,000 - £38,399	2.30 (1.03, 5.12)*	2.30 (1.01, 5.25)*	2.47 (1.03, 5.92)**
	More than £38,399	3.32 (1.62, 6.78)**	3.31 (1.57, 6.98)**	3.62 (1.60, 8.16)**
At least twice per day	Less than £16,100	-	-	-
	£16,100 - £21,249	0.88 (0.39, 1.94)	0.87 (0.38, 1.99)	0.94 (0.42, 2.11)
	£21,250 - £27,999	1.78 (0.83, 3.84)	1.69 (0.76, 3.73)	2.16 (0.94, 4.97)
	£28,000 - £38,399	3.95 (1.85, 8.42)***	3.69 (1.69, 8.02)**	4.90 (2.13, 11.29)***
	More than £38,399	6.35 (3.21, 12.57)***	6.01 (2.95, 12.23)***	8.04 (3.65, 17.69)***
**Children in household**				
Once per day	One child	-	-	
	Two children		0.62 (0.34, 1.11)	
	Three children or more		1.08 (0.53, 2.19)	
At least twice per day	One child		-	
	Two children		0.75 (0.44, 1.29)	
	Three children or more		0.84 (0.42, 1.67)	
**Adults in household**				
Once per day	Single adult			-
	Two adults			0.86 (0.44, 1.68)
	Three adults or more			0.96 (0.32, 2.84)
At least twice per day	Single adult			-
	Two adults			0.67 (0.35, 1.26)
	Three adults or more			0.45 (0.15, 1.36)

Logistic regression models for household demographic characteristics and fresh fruit and vegetable consumption, OR (95% CI).

*** <0.001

** <0.01

* <0.05.

**Table 3:** Demographic characteristics and processed food consumption.

More than once per week (base: less than once per week)	Model 2.a	Model 2.b	Model 2.c
**Annual household income**			
Less than £16,100	-	-	-
£16,100 - £21,249	0.87 (0.45, 1.67)		
£21,250 - £27,999	0.64 (0.33, 1.23)		
£28,000 - £38,399	0.94 (0.52, 1.70)		
More than £38,399	0.83 (0.49, 1.41)		
**Children in household**			
One child	-	-	-
Two children		1.07 (0.73, 1.57)	
Three children or more		1.52 (0.93, 2.48)	
**Adults in household**			
Single adult	-	-	-
Two adults			0.85 (0.56, 1.27)
Three adults or more			0.81 (0.37, 1.79)

Logistic regression models for household demographic characteristics and processed food consumption, OR (95% CI).

*** <0.001

** <0.01

* <0.05.

**Table 4:** Food insecurity, food bank use and fresh fruit and vegetable consumption.

Fresh fruit and vegetable consumption base: three times per week or less		Model 3.a	Model 3.b	Model 3.c	Model 3.d	Model 3.e
**Food insecure**						
Once per day	No	-	-		-	-
	Yes	0.46 (0.28, 0.76)**	0.64(0.36, 1.13)		0.52 (0.31, 0.88)**	0.69 (0.38, 1.24)
At least twice per day	No	-	-		-	-
	Yes	0.23 (0.14, 0.37)***	0.40 (0.23, 0.69)**		0.26 (0.16,0.43)***	0.42 (0.24, 0.75)**
**Annual household income**						
Once per day	Less than £16,100		-			-
	£16,100 - £21,249		0.86 (0.38, 1.97)			0.80 (0.34, 1.85)
	£21,250 - £27,999		0.81 (0.33, 1.95)			0.66 (0.27, 1.64)
	£28,000 - £38,399		1.74 (0.73, 4.14)			1.44 (0.59, 3.50)
	More than £38,399		2.24 (1.00, 5.00)*			1.93 (0.84, 4.39)
At least twice per day	Less than £16,100		-			-
	£16,100 - £21,249		0.72 (0.31, 1.67)			0.66 (0.28, 1.56)
	£21,250 - £27,999		1.29 (0.57, 2.93)			1.06 (0.45, 2.46)
	£28,000 - £38,399		2.50 (1.09, 5.71)*			2.03 (0.86, 4.75)
	More than £38,399		3.52 (1.63, 7.62)**			3.02 (1.37, 6.68)**
**Food bank use**						
Once per day	No					-
	Yes			0.33 (0.15, 0.72)**	0.45 (0.20, 1.01)	0.44 (0.18,1.04)
At least twice per day	No					-
	Yes			0.24 (0.12, 0.49)***	0.41 (0.19, 0.90)*	0.43 (0.19,1.00)

Logistic regression models for food insecurity status, food bank use and fresh fruit and vegetable consumption, OR (95% CI).

*** <0.001

** <0.01

* <0.05.

**Table 5:** Food insecurity, food bank use and processed food consumption.

	Model 4.a	Model 4.b	Model 4.c	Model 4.d
**Food insecure**				
No	-		-	-
Yes	1.13 (0.77, 1.66)			0.98 (0.62, 1.54)
**Food bank use**				
No		-	-	-
Yes		2.67 (1.41,5.05)**	2.75 (1.39, 5.45)**	2.68 (1.33, 5.37)**
**Annual household income**				
Less than £16,100			-	-
£16,100 - £21,249			0.91 (0.46, 1.78)	0.92 (0.46, 1.84)
£21,250 - £27,999			0.78 (0.40, 1.53)	0.79 (0.39, 1.60)
£28,000 - £38,399			1.18 (0.64, 2.18)	1.22 (0.63, 2.36)
More than £38,399			1.00 (0.58, 1.72)	0.98 (0.53, 1.81)

Logistic regression models for food insecurity status, food bank use and processed food consumption, OR (95% CI).

*** <0.001

** <0.01

* <0.05.

## Data Availability

York Research Database: Food insecurity and food aid in York, https://doi.org/10.15124/1c916cfe-1cbc-46e4-a2ed-3064094725ac^37^. This project contains the following underlying data: - De-identified survey data (n=612) - De-identified qualitative data for question 8 of the survey York Research Database: Food insecurity and food aid in York, https://doi.org/10.15124/1c916cfe-1cbc-46e4-a2ed-3064094725ac^37^. This project contains the following extended data: - Copy of the survey - Topic guide for the focus groups Data are available under the terms of the Creative Commons Attribution 4.0 International license (CC-BY 4.0). The data derived from the focus groups informs two further publications, a journal article (under review) and a monograph, published with Policy Press in 2022. In line with our data sharing agreement, these de-identified data will be made available on an open access basis following these two publications. In the interim, these data can be made available on request to the first author (madeleine.power@york.ac.uk) to bona fide researchers who provide information regarding proposed use. Quotes throughout the article reflect the content of the focus group and survey data.
